# Reversible cerebral vasoconstriction syndrome–related headache and delayed cerebral infarction: a mini review

**DOI:** 10.3389/fnins.2025.1698056

**Published:** 2025-10-21

**Authors:** Xinxin Zhang, Xu He, Jieying Zhang, Qian Zhu, Pukai Jin, Jin Yang

**Affiliations:** ^1^National Clinical Research Center for Chinese Medicine Acupuncture and Moxibustion, Tianjin, China; ^2^First Teaching Hospital of Tianjin University of Traditional Chinese Medicine, Tianjin, China; ^3^HeCares Integrative Medicine Center, Sunnyvale, CA, United States; ^4^ProCare Acupuncture and Wellness, Fairfax, VA, United States

**Keywords:** reversible cerebral vasoconstriction syndrome, thunderclap headache, delayed cerebral infarction, watershed infarct, angiography, calcium-channel blocker

## Abstract

Reversible cerebral vasoconstriction syndrome is a major cause of thunderclap headache and a preventable source of delayed ischaemic stroke. Despite expanding recognition, diagnosis is often delayed because early neuroimaging may be normal and vasoconstriction peaks in weeks two to three, and management remains experience-based rather than trial-anchored. In this mini-review we summarize advances in clinicoradiological definition and pathophysiology of tone dysregulation, outline risk-stratified diagnostic pathways built on serial CTA/MRA with confirmatory DSA when needed, high-resolution vessel-wall MRI to exclude inflammatory arteriopathy, perfusion MRI/CT and arterial spin labeling, and bedside transcranial Doppler, and appraise translational opportunities spanning time-anchored surveillance, perfusion-preserving care bundles and pragmatic endpoints. We also discuss enduring challenges—including nosological overlap with primary CNS vasculitis, non-standardized imaging schedules, heterogeneous blood-pressure targets and a paucity of randomized data—that temper implementation. By integrating time-aware vascular and perfusion readouts (e.g., planned week-2–3 repeat angiography, ASL hypoperfusion mapping, sustained Doppler velocities) with trigger withdrawal, cautious blood-pressure management and symptomatic vasodilators such as calcium-channel blockers and magnesium in selected contexts, emerging strategies aim to preserve cerebral perfusion, anticipate delayed infarction and standardize follow-up across settings. Our synthesis provides an appraisal of the evolving landscape of RCVS care and outlines pragmatic standards and avenues for prospective evaluation. We hope these insights will assist researchers and clinicians as they endeavor to implement more effective, individualized regimens.

## 1 Introduction

RCVS is defined by recurrent thunderclap headache (peak ≤1 min; attacks typically last ≥5 min), smooth segmental narrowing across ≥2 intracranial territories, and angiographic reversibility by ≈12 weeks after exclusion of aneurysmal SAH, vasculitis, dissection, and infection (Cho et al., 2020; Wu et al., 2021; Chen and Wang, 2022). The disease course is dynamic; initial neuroimaging can be normal and vasoconstriction typically reaches maximum severity about 2 to 3 weeks after symptom onset, a window that coincides with the period of greatest risk for ischemic complications (Chen et al., 2021; John et al., 2016; Perillo et al., 2022; Kano et al., 2021). These features render RCVS a leading cause of sudden severe headache with potential for delayed cerebral infarction despite an initially reassuring evaluation, underscoring the need for time-aware diagnostic pathways and follow-up imaging.

Current evidence supports a multifactorial pathophysiology centered on transient dysregulation of cerebrovascular tone, in which sympathetic overactivity, endothelial dysfunction, and blood–brain barrier perturbation interact with exogenous and physiological triggers (Cho et al., 2021; Sequeiros et al., 2020; Edjlali et al., 2020; Ospel et al., 2020). Common precipitants include exposure to vasoactive or serotonergic/sympathomimetic agents, abrupt catecholaminergic surges related to exertion or Valsalva, and the peripartum state; these factors likely lower the threshold for diffuse, migrating vasoconstriction that begins distally and progresses proximally (Pensato et al., 2020; Su et al., 2023; Patel et al., 2021). This mechanistic frame provides a rationale for the characteristic headache phenotype and for the observed temporal dissociation between early hemorrhagic events and later ischemic injury.

The neuroradiologic spectrum spans early cortical convexity subarachnoid hemorrhage and posterior reversible encephalopathy–pattern edema to watershed-predominant infarction emerging days to weeks after presentation. Magnetic resonance imaging may be unrevealing initially and later demonstrate watershed or border-zone infarcts; angiography (CTA/MRA/DSA) shows the characteristic “string-of-beads” pattern with smooth, multisegmental narrowing and interposed dilatations, and repeat vascular imaging during the second–third week is often decisive given the delayed peak in vasoconstriction (Arandela et al., 2021; Mansoor et al., 2021; Li et al., 2023; Lange et al., 2023). These observations explain why patients with RCVS-related thunderclap headache can subsequently develop delayed cerebral infarction despite early negative studies and emphasize the importance of structured surveillance strategies.

Against this background, the present mini-review focuses on RCVS-related headache as the sentinel clinical event and delineates the links to delayed cerebral infarction, with attention to phenotype definition, mechanistic hypotheses, risk-stratified diagnostics, and therapeutic implications consistent with contemporary evidence.

## 2 Clinical–radiographic phenotype of RCVS-related headache

RCVS-related headache is an abrupt, severe thunderclap pain peaking in ≤1 min (ICHD-3), lasting ≥5 min (often minutes–hours), and recurring over days–weeks; “recurrent TCH” denotes ≥2 such attacks ≥24 h apart within ~1 month (Tentolouris-Piperas et al., 2023; Kim et al., 2025; Nelson, 2024). Vasoactive and sympathomimetic exposures and the peripartum state are common contexts, supporting a syndrome of transient dysregulation of cerebrovascular tone (Isikbay et al., 2021; Liu et al., 2024; Magid-Bernstein et al., 2021). Angiographic reversibility within roughly 3 months remains a defining criterion and reflects the self-limited course of vasoconstriction.

Early brain imaging can be unrevealing. As the vasculopathy evolves, vascular studies show smooth, segmental multiterritorial narrowing with interposed dilatations (“string-of-beads”), while parenchymal MRI may transition from normal to complications that cluster in two temporal phases (Ribas et al., 2023; Sivagurunathan et al., 2024; Ramaswamy et al., 2025; Spadaro et al., 2021). Hemorrhagic events—most characteristically small cortical convexity subarachnoid hemorrhage—tend to occur near onset, whereas ischemic complications often arise later, frequently with border-zone/watershed predilection; posterior reversible encephalopathy–pattern vasogenic edema may co-occur (Singhal, 2023; Lange et al., 2025; Hostettler et al., 2025). These features map the dynamic coupling of headache and vessel caliber change and explain why repeat vascular imaging after the first 1 to 3 weeks is often decisive when initial studies are non-diagnostic.

The angiographic phenotype is captured across CTA, MRA, and (when required) catheter DSA. Transcranial Doppler can demonstrate diffusely elevated flow velocities corresponding to vasospasm and may be used to monitor the trajectory, while arterial spin labeling and other perfusion techniques can demonstrate hypoperfusion during the active phase (Edlow et al., 2025; Favrelière et al., 2024; Nakaya et al., 2024). These modalities are complementary rather than diagnostic in isolation; the combination of clinical thunderclap headache, evolving multifocal vasoconstriction, and subsequent reversibility underpins case definition.

High-resolution vessel-wall MRI provides important discrimination from primary angiitis of the CNS. In RCVS, mural enhancement is typically absent/minimal and non-concentric, whereas PACNS usually shows avid, concentric enhancement; 3 T black-blood pre−/post-contrast sequences are preferred, and common false positives include atherosclerosis/vasa vasorum and subacute dissection (Verma et al., 2024; dit Beaufils et al., 2024). Clinically, the headache itself sometimes migrates in location in parallel with distal-to-proximal propagation of vasoconstriction inferred on serial angiography. Transient focal symptoms, seizures, or visual disturbance may accompany attacks, but the key message for practice is temporal coupling: a normal early MRI or angiogram does not exclude RCVS, and structured follow-up imaging is required during the second to third week after onset to detect peak narrowing and to risk-stratify for delayed cerebral ischemia. Table 1 summarizes phase-specific operational descriptors that link the headache phenotype to radiographic findings and the preferred modalities used to detect them.

**Table 1:** Operational descriptors of the clinical–radiographic phenotype of RCVS-related headache across disease phases.

Phenotypic domain	Core descriptors	Typical timing from index thunderclap	Modality that best detects	Practical implication
Headache onset and pattern	Thunderclap pain peaking within seconds; recurrent over days–weeks; possible migratory location	Day 0–21	Clinical history; standardized headache diary	Defines sentinel event; guides timing of repeat imaging
Early hemorrhagic correlates	Cortical convexity subarachnoid hemorrhage; less often intraparenchymal hemorrhage	Day 0–7	Non-contrast CT; SWI/T2 MRI	Supports diagnosis; prompts BP control and hemorrhage monitoring
Vasogenic edema	PRES-like posterior-predominant edema; reversible with time	Day 0–14	MRI (FLAIR, DWI/ADC)	Indicates blood–brain barrier stress; avoid precipitants that raise BP
Evolving vasoconstriction	Smooth, multisegmental narrowing with interposed dilatations in multiple territories	Peaks ~week 1–3	CTA/MRA; DSA if ambiguity persists	Repeat imaging is decisive when baseline studies are normal
Delayed ischemia	Border-zone/watershed-predominant infarcts; small cortical DWI lesions possible	Day 3–21	MRI (DWI/ADC); perfusion MRI/CT	Triggers escalation of monitoring and targeted secondary prevention
Flow physiology	Diffusely elevated flow velocities consistent with vasospasm	Day 1–21	Transcranial Doppler	Bedside trend tracking of vasoconstriction burden
Vessel-wall features	Absent/minimal, often non-concentric mural enhancement; luminal change disproportional to wall signal	Day 1–21	High-resolution vessel-wall MRI	Helps differentiate from vasculitis and other arteriopathies
Reversibility	Resolution of vasoconstriction on follow-up	By ≈12 weeks	CTA/MRA (follow-up)	Confirms diagnosis and anchors endpoint for care pathway

PRES, posterior reversible encephalopathy syndrome; CT, computed tomography; MRI, magnetic resonance imaging; CTA, CT angiography; MRA, MR angiography; DSA, digital subtraction angiography; SWI, susceptibility-weighted imaging; DWI/ADC, diffusion-weighted imaging/apparent diffusion coefficient.

## 3 Mechanistic links to delayed cerebral infarction

**Figure 1:**
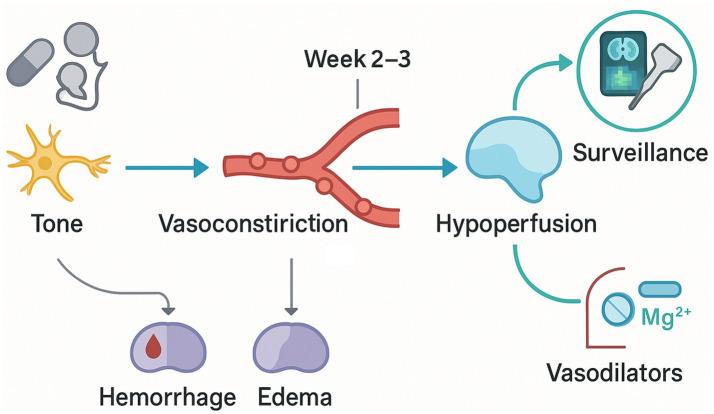
Time-anchored mechanistic cascade and care pathway in RCVS.

As shown in Figure 1, the temporal dissociation between the initial thunderclap headache and later ischemic injury in reversible cerebral vasoconstriction syndrome can be explained by a cascade in which dysregulated cerebrovascular tone evolves from transient, predominantly distal arterial narrowing to a multiterritorial vasoconstrictive state that peaks during the second to third week after symptom onset (Sugita et al., 2025; You et al., 2023; Jeanneret et al., 2022). Sympathetic overactivity and endothelial dysfunction appear to be the dominant upstream drivers of this dynamic, reducing vasodilatory reserve and impairing autoregulatory responses to fluctuations in systemic blood pressure. As vasoconstriction intensifies and propagates across vascular territories, resting cerebral blood flow can be maintained at the cost of exhausted reactivity; once further challenged—by posture, Valsalva, nocturnal dips, or antihypertensive exposure—regional perfusion falls below ischemic thresholds (Van Ly et al., 2023; Vranic et al., 2021; Lindner et al., 2023). This time-linked fall in perfusion reserve aligns with the recognized window of highest ischemic risk and provides a mechanistic basis for delayed infarction despite normal early imaging.

Converging radiographic data support a hemodynamic mechanism. Diffusion-weighted imaging frequently reveals infarcts with a watershed/border-zone predilection, often bilateral, consistent with selective vulnerability of terminal fields under conditions of global or multiterritorial vasoconstriction (Osmont et al., 2024; Li et al., 2024; Singhal, 2023). Perfusion techniques can demonstrate reduced cerebral blood flow with prolongation of mean transit time in affected regions during the active phase, and case-based perfusion CT has shown hypoperfusion concordant with contemporaneous segmental narrowing on angiography (Kim et al., 2025; Wang et al., 2024; Kang et al., 2024). The border-zone preference accords with established principles of flow failure in distal arterial territories where collateral supply is limited, thereby linking the spatial pattern of infarction to the physiology of vasoconstriction-mediated hypoperfusion.

The biphasic complication profile—early cortical convexity subarachnoid hemorrhage and vasogenic edema followed by later ischemia—can be integrated within this framework. In the early phase, transient surges in pressure and flow in the setting of endothelial dysfunction and blood–brain barrier stress favor hemorrhagic and edema-dominant complications; as vasoconstriction intensifies and autoregulation remains impaired, the balance shifts toward flow-dependent tissue injury in watershed territories (Oliveira et al., 2022; Kaufmann et al., 2024; Garg et al., 2021). Overlap with posterior reversible encephalopathy syndrome in both triggers and imaging features reflects shared vascular dysregulation rather than distinct disease processes, reinforcing the concept that delayed infarction in reversible cerebral vasoconstriction syndrome is primarily the result of evolving tone-mediated hypoperfusion superimposed on a compromised microvascular interface. This mechanistic model substantiates surveillance during the second–third week after onset and supports perfusion-sensitive monitoring as a means to anticipate and mitigate delayed cerebral infarction.

## 4 Diagnostic trajectory and risk stratification

The diagnostic trajectory for suspected reversible cerebral vasoconstriction syndrome should be explicitly time-aware and anchored in the dynamic evolution of vasoconstriction, because early structural and vascular studies can be normal while arterial narrowing peaks during the second to third week after onset with subsequent angiographic reversibility by approximately 12 weeks (Garg et al., 2021; Chen et al., 2024; Utukuri et al., 2023). Initial evaluation should include non-contrast head CT to exclude aneurysmal subarachnoid hemorrhage, baseline parenchymal MRI with diffusion-weighted imaging, and vascular imaging with CTA or MRA. When clinical suspicion remains high despite non-diagnostic studies, interval repeat vascular imaging between days 7 and 21 is essential to capture the delayed apex of multifocal, smooth segmental narrowing and to establish reversibility on follow-up, consistent with contemporary criteria (Song et al., 2024; Huang et al., 2024; Hoffmann et al., 2025). Algorithm: Day 0 CTA/MRA ± MRI; planned repeat vascular imaging at days 7–21; follow-up at ~12 weeks to confirm reversibility; re-image sooner for new deficits or Doppler velocity surges. This staged approach is supported by the foundational clinical–radiologic description and temporal criteria consolidated in major reviews and is critical to prevent false reassurance from early-negative imaging.

Digital subtraction angiography remains the reference method when noninvasive angiography is equivocal or when clinical deterioration occurs; it characteristically shows multiterritorial “string-of-beads”–type alternating constrictions and dilatations. However, because DSA is invasive and not universally required, CTA and MRA are preferred first-line modalities with planned repetition to detect progression and subsequent recovery (Kharal et al., 2024; Asawavichienjinda et al., 2024; Rozen and Bhatt, 2022). The choice to proceed to DSA should be individualized based on evolving deficits, extent of vessel involvement on noninvasive studies, and the need to exclude alternative vasculopathies.

Complementary physiologic assessments refine early diagnosis and risk stratification. Arterial spin labeling (prefer pCASL) should use adult PLDs around 2.0–2.5 s—or multi-PLD when transit is delayed—because slow arterial transit can otherwise mimic hypoperfusion; these perfusion data highlight territories at risk before diffusion changes. In emergency and monitoring contexts, transcranial Doppler or transcranial color-coded Doppler can quantify elevated flow velocities that parallel the clinical course, offering a practical, bedside method to track vasoconstriction intensity and resolution (Bonura et al., 2023; Montarello et al., 2024; Alapatt et al., 2021). Integrating perfusion-sensitive MRI (or CT perfusion) and Doppler surveillance provides a functional correlate to luminal change and should escalate monitoring—even if angiography is nondiagnostic—when perfusion remains abnormal.

Differentiation from primary angiitis of the central nervous system is a recurrent diagnostic challenge with direct therapeutic consequences. High-resolution vessel-wall MRI supports this distinction: RCVS typically shows absent or minimal, non-concentric mural enhancement, whereas central nervous system vasculitis more often demonstrates robust, concentric vessel-wall enhancement (Zeitouni et al., 2021; Aibani et al., 2025; Erhart et al., 2023). When the clinical picture is atypical or when inflammatory arteriopathy remains in the differential, adding vessel-wall imaging to serial luminal angiography increases diagnostic confidence without delaying surveillance for the expected evolution of RCVS.

Risk stratification should also incorporate RCVS-focused scores: RCVS2 (≥5 “probable”, 3–4 “possible”, ≤2 unlikely) and the RCVS–TCH score (derived for TCH cohorts); use higher scores to intensify serial imaging, noting RCVS2 is less useful in undifferentiated TCH-only triage. Observational data indicate that ischemic stroke complicates a relevant subset of RCVS presentations and clusters within the same 2–3-week window in which vasoconstriction peaks; conventional cerebrovascular comorbidities appear to increase susceptibility to ischemic injury once vasodilatory reserve is exhausted (Liang et al., 2024; Chen and Wang, 2022; Kunitake et al., 2022). In parallel, cohort analyzes of overall complications describe demographic and exposure-related variables that associate with focal deficits or demonstrable brain lesions during the course, including increasing age, postpartum status, and treatment with serotonergic antidepressants; surges in blood pressure and the absence of prototypical thunderclap headache at onset have also been implicated. Although these studies differ in endpoints and design, they converge on the principle that patient factors and precipitating contexts modulate risk on top of the hemodynamic substrate (Jung et al., 2025; Abu-Abaa et al., 2022; Okazaki et al., 2025). These insights justify tiered follow-up: patients with high-risk clinical features or with progressive multiterritorial narrowing, Doppler evidence of sustained high velocities, or perfusion deficits should undergo closer inpatient or early outpatient surveillance with expedited repeat angiography and perfusion assessment, whereas patients lacking these features may be followed on a standard schedule provided that clinical status remains stable.

Radiographic pattern recognition contributes additional prognostic information. Infarcts, when present, often display wedge-shaped, border-zone distributions consistent with flow failure in terminal fields under conditions of diffuse vasoconstriction, a pattern that reinforces the hemodynamic origin of delayed ischemia and supports the use of perfusion-sensitive monitoring during the active phase (Collins et al., 2023; Patel et al., 2023; Madapoosi et al., 2024). Identification of this predilection should prompt targeted review of internal and cortical watershed territories on serial MRI and careful blood pressure management to avoid iatrogenic reductions in cerebral perfusion pressure while vasoreactivity is impaired.

A time-structured diagnostic pathway and a multidomain risk framework—encompassing clinical precipitants and demographics, serial luminal and wall imaging, and perfusion or Doppler indicators—provide a coherent strategy to confirm RCVS, differentiate it from inflammatory vasculopathies, and pre-empt delayed cerebral infarction. This strategy operationalizes routine early noninvasive angiography with scheduled repetition at 1–3 weeks, selective DSA when necessary, adjunctive vessel-wall MRI for inflammatory differentials, and physiologic monitoring to identify impaired reserve, thereby aligning diagnostic confirmation with prevention-oriented risk management.

## 5 Therapeutic strategies and future directions

Therapeutic strategies for reversible cerebral vasoconstriction syndrome should be organized around three principles that emerge consistently from the clinicoradiologic trajectory: removal of precipitating factors, protection of perfusion during the peak vasoconstrictive window, and time-structured surveillance to detect treatable deterioration. Withdrawal of vasoactive triggers and meticulous blood pressure management remain foundational. Because vasoconstriction peaks at weeks 1–3, avoid SBP < 110–120 mmHg or >20% acute drops and minimize nocturnal dips; with early convexity SAH, target SBP ~ 120–140 mmHg initially; in postpartum RCVS, align thresholds with obstetric guidance (treat ≥160/110; goal <150/100) and consider magnesium (McGraw et al., 2025; Girfanova et al., 2025; Cho et al., 2025). Oral calcium-channel blockers are symptomatic: nimodipine 30–60 mg PO q4h (start lower if hypotensive; CYP3A4 interactions), verapamil SR 120–240 mg/day PO (headache relief commonly reported), with selective IV/IA verapamil rescue; taper over ~2–4 weeks as symptoms/velocities improve; prevention of stroke remains unproven (Mitsutake et al., 2025; Sreedharan et al., 2025; Madapoosi et al., 2025). Intravenous magnesium may be used (esp. peripartum): 4–6 g IV load over 20–30 min, then 1–2 g/h for ~24 h with reflex/renal monitoring; evidence favors headache relief over stroke prevention (Verma et al., 2024; dit Beaufils et al., 2024; Zeitouni et al., 2021; Erhart et al., 2023). Antiplatelet or anticoagulant therapy is not routinely recommended in the absence of another indication given the frequency of early hemorrhagic correlates; seizure prophylaxis is reserved for patients with clinical events or high-risk imaging features, with preference for short courses aligned to the active phase. When neurological deficits progress in parallel with angiographic worsening despite conservative measures, escalation with endovascular vasodilators can be considered in carefully selected cases; such rescue strategies may transiently improve caliber and perfusion yet require integration with the overarching, time-aware plan because durability is uncertain and the invasive risk is nontrivial.

Future directions should convert this pragmatic bundle into testable, time-anchored care pathways and prioritize outcomes that reflect RCVS biology. Prospective studies should randomize early, protocolized calcium-channel blockade plus standardized trigger withdrawal and blood-pressure targets against optimized supportive care, with adaptive enrichment for patients at greatest ischemic risk defined by progressive multiterritorial narrowing, sustained transcranial Doppler velocities, or perfusion abnormalities on arterial spin labeling or CT/MR perfusion (Edlow et al., 2025; Nakaya et al., 2024). Composite endpoints that pair infarction-free survival through week three with validated headache and functional measures are more appropriate than isolated angiographic metrics and would align therapeutic assessment with the recognized risk window (Sugita et al., 2025; Madapoosi et al., 2024). Given the frequency of diagnostic ambiguity early in the course, trials should embed vessel-wall MRI to exclude inflammatory arteriopathies and mandate repeat luminal imaging in week one to three to capture peak narrowing and document reversibility, ensuring that treatment effects are not confounded by misclassification (Song et al., 2024; Huang et al., 2024; Hoffmann et al., 2025; Zeitouni et al., 2021; Aibani et al., 2025; Erhart et al., 2023). Ambulatory blood-pressure monitoring and nocturnal hemodynamic profiling warrant evaluation as adjuncts to prevent perfusion dips during impaired autoregulation; protocolized avoidance of vasodilatory reserve depletion—such as timing of antihypertensives, analgesics, and exertion—could be tested as a low-risk preventive strategy during the peak phase. Registries with prespecified data elements should clarify subgroup responses in postpartum, drug-exposed, and comorbidity-laden populations and inform risk calculators that integrate clinical context with serial angiography, perfusion imaging, and Doppler indices to triage intensity and duration of monitoring (Liang et al., 2024; Okazaki et al., 2025). Rescue endovascular therapy requires harmonized technical standards, predefined physiological entry criteria, and core-lab adjudication of perfusion response to establish when potential benefit outweighs risk and how best to coordinate such procedures with systemic vasodilators and blood-pressure targets. These steps would transform a heuristic, experience-based management paradigm into reproducible pathways that are synchronized to the temporal physiology of RCVS, minimize delayed cerebral infarction, and standardize decision-making across centers while preserving individualized care.
